# Breastfeeding Practices in Relation to Country of Origin Among Women Living in Denmark: A Population-Based Study

**DOI:** 10.1007/s10995-014-1486-z

**Published:** 2014-04-19

**Authors:** Marianne Busck-Rasmussen, Sarah Fredsted Villadsen, Filippa Nyboe Norsker, Laust Mortensen, Anne-Marie Nybo Andersen

**Affiliations:** Section of Social Medicine, Department of Public Health, University of Copenhagen, Oster Farimagsgade 5, Box 2099, 1014 Copenhagen K, Denmark

**Keywords:** Breastfeeding, Migrants, Acculturation, Health care disparity, Epidemiology

## Abstract

The objective of this study was to describe breastfeeding practices and to compare the risk of suboptimal breastfeeding of women living in Denmark according to country of origin, and further to examine how socio-economic position and duration of stay in the country affected this risk. Information on breastfeeding of 42,420 infants born 2002–2009 and living in eighteen selected Danish municipalities was collected from the Danish Health Visitor’s Child Health Database. The data was linked with data on maternal socio-demographic information from Danish population-covering registries. Suboptimal breastfeeding was defined as <4 months of full breastfeeding as described by the Danish Health and Medicines Authority. We used logistic regression to model the crude associations between suboptimal breastfeeding and country of origin, and taking maternal age and parity, and a variety of parental socio-economic measures into account. Suboptimal breastfeeding was more frequent among non-Western migrant women than among women of Danish origin. Women who were descendants of Turkish and Pakistani immigrants had a higher risk of suboptimal breastfeeding as compared to the group of women who had migrated from the same countries, suggesting that acculturation did not favor breastfeeding. For all but the group of women who had migrated from Pakistan, adjustment for socio-demographic indicators (age, parity, education, attachment to labour market, and income) eliminated the increased risk of suboptimal breastfeeding. There was no evidence for differences in the breastfeeding support provided at hospital level according to migrant status. Suboptimal breastfeeding was more frequent among women who were non-Nordic migrants and descendants of migrants than among women with Danish origin.

## Introduction

The beneficial effect of breastfeeding on infant health is well described, and WHO recommends at least 6 months of exclusive breastfeeding for children globally [1]. One of the most important short term effects of breastfeeding is the protection against infectious diseases, and breastfed infants have been demonstrated to have lower prevalence of inflammatory bowel diseases, childhood cancers, and type-1 diabetes [2]. Breastfed children have been shown to have improved cognitive performance [3–5], while the effects on overweight and obesity are uncertain [4, 6].

Women’s decision and ability to breastfeed are affected by their social context. In many western countries are young maternal age and poor socio-economic position risk factors of no initiation and short duration of breastfeeding [7–10]. However, the influence of socio-demographic parameters on breastfeeding might differ between societies. Studies from countries in Western Europe and the US have shown that breastfeeding frequencies are higher among migrant women than in the native populations [11–14]. It seems that with acculturation, measured by years of residency in the new country breastfeeding prevalences decline and, likewise, first generation descendants of migrants breastfeed less than the women who migrated [11, 14]. A Swedish study reported that there were no ethnic differences in breastfeeding at 6 months, while more immigrant mothers continued breastfeeding until at least 12 months [9].

In Denmark, breastfeeding initiation and continuation of full breastfeeding in 4 months is high, 95 and 60 %, respectively [15]. The national recommendations are full breastfeeding for 6 months, but the guidelines acknowledge that some children will be ready for complementary feeding after 4 months. Further, partial breastfeeding is recommended to continue until at least 12 months [16]. At the international level WHO and UNICEF have encouraged breastfeeding with the *Baby Friendly Hospital Initiative* [17] that has set guidelines for breastfeeding support at health facilities, including: not giving formulas (unless medically indicated) and to help the mothers initiate breastfeeding within one-half hour of birth [18]. In Denmark, additional support for breastfeeding is offered to all newborns and their mothers at home visits where health visitors (specially trained nurses) after delivery assist the family in ensuring the well-being of the child. Unfortunately, no official statistics are available, but the general understanding among leaders of this system and the authors’ experience are that the health visitors are widely accepted and used by the large majority (more than 95 %) of the families, including families with migrant background.

Breastfeeding practices among migrants have scarcely been studied in Denmark. A single study has described the breastfeeding prevalence as lower in migrant women than in women of Danish origin [15]. However, in this study country of origin was not taken into account. It has been shown that reproductive and child health are diverging among the different migrant groups in Denmark [19, 20], and to understand the mechanisms behind the potential increased vulnerability of the migrant groups, it is important not to treat migrants as one entity. Therefore, in order to guide future preventive strategies, we find it justified that a better understanding of patterns in breastfeeding prevalence and the mechanisms behind will be obtained using a country of origin approach. The objective of this study was to assess the risk of suboptimal breastfeeding among groups of migrant women as compared to women of Danish origin, and to describe how measures of socioeconomic position, acculturation and breastfeeding support affect these associations.

## Materials and Methods

### The Danish Health Visitor’s Child Health Database

The Danish Health Visitor’s Child Health Database was established by the municipalities in the county of Copenhagen in 2002 with the aims of documentation and monitoring of infant health and the work of municipality-based health visitors. All health visitors in 18 municipalities provided data to the database during the period 2002–2009: ten municipalities participated during the total period, the remaining during one or more of the years. The database encompasses information on 47,869 infants (corresponding to more than 90 % of the children born in the municipalities) that had at least one health visit during the period of coverage.

The data on breastfeeding stem from a standardized systematic registration file for every infant, kept by the health visitors who recorded details on the child, the family and service provided by the health visitors during the first year of life [15]. From the same data source we used health visitor registered information about maternal parity (categorized as 1, 2, and 2+), and a dichotomous indication of whether the newborn was put to the mothers’ breast within 2 h of birth (yes/no) and whether the newborn was given formula during the stay at hospital after birth (yes/no). The latter two variables were registered by the health visitor at first visit after birth as indicators of breastfeeding support, as these are two of the Baby Friendly Hospital Initiative ten steps to successful breastfeeding [18]. The health visitors also registered duration of full breastfeeding, defined as age until which the infant was exclusively given breast milk, supplemented only by water or at maximum one meal of formula per week. This information was used to establish the main outcome of interest in the study: full breastfeeding until the age of at least 4 months (yes/no).

### Socio-Demographic Data from Statistics Denmark

From the Population Registry in Statistics Denmark we obtained information on maternal and paternal unique personal identification number (PIN) that, beside carrying information about the year of birth for the mother, served as key variable for all linkages. For women born outside Denmark, country of origin and year of immigration were also obtained. Maternal age was categorized in four strata (<25, 25–29, 30–34, and 35+ years). From year of immigration, maternal year of birth, and infant year of birth we calculated maternal age at migration to Denmark (<10, 10–18, >18 years) and number of years lived in Denmark before delivery (<10 years, 10+ years). From the Integrated Database for Labour Market Research at Statistics Denmark we obtained information about maternal and paternal educational level (recoded from ISCED to <10, 10–12 and >12 years of formal education), maternal and paternal attachment to labour market, dichotomized into being in work (having paid work more than 50 % of the year, for mothers the calendar year before birth, for fathers in the calendar year of birth) and out of work, and, finally, household income (sum of maternal and paternal disposable income) calculated into percentiles of the study population born in the same year and categorized in five strata (0–5, 6–24, 25–49, 50–74, and 75–100 %) of which the two first groups represent the quartile with the lowest income.

### Country of Origin

We used the available data in Statistics Denmark’s regarding ethnicity to determine maternal country of origin: The country of origin is *Danish* if (at least) one parent is born in Denmark *and* has Danish citizenship. *Migrants* are born outside Denmark and none of the parents of a migrant is born in Denmark *and* has Danish citizenship. For migrants, the country of origin is the same as the country of birth. *Descendants* are born in Denmark and none of the parents of a descendant is born in Denmark and has Danish citizenship. A child born of two descendants with non-Danish citizenship is also classified as a descendant. For descendants, the country of origin is defined as country of citizenship. If maternal and paternal country of birth and citizenship are different and not Danish, country of origin is defined based on maternal country of birth and citizenship. As follows from this description, Danish citizenship is not obtained automatically by virtue of birth in the country if the parents are non-Danish citizens and double citizenship is not allowed. Citizenship can be obtained after application in a relatively complicated procedure.

### Study Population

The study population was grouped according to maternal country of origin and only groups that exceeded 250 individuals in the database were included.

Of the 47,869 infants in the Danish Health Visitor’s Child Health Database, 986 (2.1 %) were excluded due to missing information on PIN-number, 1,044 (2.2 %) had no information about country of origin in the Population Registry, and 3,410 (7.1 %) had a country of birth did not fulfill the inclusion criteria. Thus, 42,420 infants were included.

### Statistical Analyses

Distributions of parental socio-demographic variables and indicators of breastfeeding support according to maternal counties of origin were cross tabulated and the proportion of infants, who were fully breastfed, was calculated according to maternal country of origin and stratified on parental characteristics.

The crude odds ratios with 95 % confidence intervals for suboptimal breastfeeding according to country of origin were estimated using logistic regression with women of Danish origin as the reference group. Analyses exploring the relation between strata of socio-demographic characteristics and maternal country of origin on the risk of suboptimal breastfeeding were estimated in logistic regression models using the most prevalent value of the characteristic among mothers of Danish origin as the reference.

The risk of suboptimal breastfeeding according to maternal country of origin was furthermore estimated in a multivariable logistic regression model adjusted for maternal age and parity and all socio-demographic variables.

### Approvals

The linkage of data from the Danish Health Visitor’s Child Health Database and data from Statistics Denmark was approved by the steering committee for the Danish Health Visitor’s Child Health Database and the Danish Data Protection Agency.

## Results

Of the 42,420 infants, 87 % had mothers of Danish origin. Among the mothers of non-Danish origin, Turkish origin was the most frequent (4.9 %), followed by mothers of Pakistani origin (2.3 %). Other maternal countries of origin were Former Yugoslavia (including Serbia, Montenegro, Croatia, Bosnia and Hercegovina, Macedonia, Kosovo and Slovenia), Iraq, Morocco, Lebanon (including Palestine), and Afghanistan. The majority of mothers of non-Danish origin were migrants themselves, though, 20–30 % of mothers with Turkish, Pakistani, Former Yugoslavian and Moroccan origin were descendants, i.e. born in Denmark of women, who migrated to Denmark.

In general, the infants of Danish and other Nordic origin were more likely to be first or second born, their mothers were older than mothers of non-Nordic origin, their parents had a longer education and were more likely to be active participants at the labour market (Table 1). The household income were unequally distributed, with more than 75 % of the infants of Iraqi, Lebanese/Palestinian, and Afghan origin born in families in the lowest income quartile. Information about full breastfeeding until the age of 4 months was missing on a substantial proportion of the infants. However, missing information on breastfeeding did not appear to depend on maternal country of origin or social and demographic characteristics (data not shown).

**Table 1:** Selected characteristics of the study population (distributions in percentages). All children seen by health visitor in 18 Danish municipalities, 2002–2009

	Maternal country of origin								
	Denmark	Turkey	Pakistan	Former Yugoslavia	Iraq	Morocco	Lebanon/Palestine	Afghanistan	Other Nordic
N = 42,420	36,899	2,091	992	729	442	292	262	266	447
Descendants of migrants (%)		24.1	29.2	23.0	0.5	19.9	5.7	0.0	13.4
*Maternal age at birth*									
<25 years	9.9	26.7	18.5	23.7	26.7	14.7	37	25.2	9.2
25–29 years	27.9	40.3	44.6	41.8	31.7	33.2	29.4	35.3	22.1
30–34 years	38.8	22.2	26.6	24.3	24	28.4	21.4	24.4	41.4
35+ years	23.5	10.2	10.4	10.2	17.7	23.6	12.2	15.0	27.3
*Maternal parity*									
1	48.8	36.8	32.5	42.5	37.0	32.9	40.0	38.7	48.7
2	36.4	30.9	29.9	36.7	29.1	28.0	21.4	29.7	34.0
3+	14.9	32.3	37.6	20.8	34.0	39.4	39.1	31.5	17.7
Missing	15.4	14.8	13.7	12.2	19.2	13.0	16.0	16.5	16.3
*Maternal educational level*									
<10 years	14.9	58.5	38.3	34.6	47.6	49.2	62.9	51.1	7.5
10–12 years	39.4	31.8	44.8	48.0	37.6	39.1	30.8	35.2	34.3
>12 years	45.7	9.7	16.9	17.4	14.8	11.7	6.3	13.7	58.1
Missing	0.5	8.6	14.7	10.3	11.1	12.3	9.5	17.7	17.2
*Paternal educational level*									
<10 years	15.7	57.0	38.2	33.9	35.5	41.4	46.1	46.1	7.0
10–12 years	45.0	32.1	40.3	51.4	37.7	41.4	32.4	32.4	43.2
>12 years	39.3	10.9	21.5	14.8	26.8	17.1	21.6	21.6	49.8
Missing	1.1	9.1	8.9	10.9	9.4	12.8	14.5	14.5	21.8
*Parental attachment to labour market*									
Mother in work	87.6	57.1	39.8	66.1	18.0	44.3	27.7	23.5	82.1
Father in work	93.9	75.9	83.2	83.4	48.3	77.8	55.3	59.6	90.1
*Household income percentile*									
<6 %	3.2	11.7	17.4	7.8	19.9	8.0	16.5	17.5	4.2
6–24 %	14.9	51.6	41.3	36.1	63.4	55.2	59.3	63.0	17.3
25–49 %	25.4	27.4	25.3	34.0	10.4	23.4	20.3	12.4	19.1
50–74 %	28.0	7.7	12.3	15.0	4.0	10.1	3.0	6.0	25.1
75–100 %	28.6	1.8	4.0	7.1	2.4	3.1	0.9	1.2	34.3
Missing	2.2	4.2	4.7	5.1	4.3	2.1	9.9	5.6	2.9
*Missing information on*									
Breastfeeding at 4 month (%)	32.7	32.1	43.3	28.0	38.5	33.9	33.6	39.5	34.7

A higher proportion of the infants with mothers of other Nordic origin were fully breastfed for at least 4 months while infants with non-Nordic origin were significantly less likely to be fully breastfed as compared to infants of Danish origin (Table 2). Mothers of Turkish and Pakistani origin displayed the lowest breastfeeding prevalence. For Danish and other Nordic infants, the likelihood of being fully breastfed increased with maternal age at birth and parity, and was higher among women with a high socio-economic position. The pattern was less clear for infants of all other migrant groups. Only the Turkish minority had a clear age-related pattern that was similar to the Danish majority and first-borns were less likely to be fully breastfed in all minorities. However, the trend with parity was weaker and only significant for the Lebanese/Palestinian minority. The strong parental educational trend for full breastfeeding found for offspring of Danish women was weaker for non-Nordic minorities. In few cases reversed and no pattern was seen for household income, where the two largest minorities, the Turkish and the Pakistani, showed opposite trends. In the same two minorities, descendants of migrants were less likely to fully breastfeeding than migrants and for migrants there was a tendency of less breastfeeding the longer the migrant had lived in Denmark before the delivery and the younger the mother had been when she immigrated to Denmark.

**Table 2:** Proportion of children who were fully breastfed for 4 months or more according to parental characteristics and maternal country of origin and according to acculturation indicators and maternal country of origin

	Maternal country of origin								
	Denmark	Turkey	Pakistan	Former Yugoslavia	Iraq	Morocco	Lebanon/Palestine	Afghanistan	Other Nordic
N = 28,441	24,842	1,420	562	525	272	193	174	161	292
Overall	61.8	42.7	45.6	50.1	50.0	49.2	45.8	57.8	71.6
*Maternal age at birth*									
<25 years	36.5	33.7	44.8	47.0	47.1	48.2	38.2	52.2	50.0
25–29 years	56.9	43.6	45.8	48.9	52.7	44.1	46.0	55.6	69.0
30–34 years	66.9	50.2	44.8	57,0	47.7	54.6	54.3	62.5	72.8
35+ years	69.8	47.1	48.1	44,0	52.2	51.2	47.6	66.7	78.3
*Maternal parity*									
1	57.3	40.6	46.3	45.6	48.9	42.0	33.3	49.2	64.7
2	64.8	43.0	44.7	50.9	46.3	45.3	40.0	60.0	75.6
3+	67.5	45.2	48.7	59.8	54.9	54.2	59.7	66.7	70.2
*Maternal educational level*									
<10 years	37.0	41.1	39.2	44.2	46.9	44.1	39.6	59.1	47.4
10–12 years	56.2	44.6	46.1	50.0	51.7	50.0	56.6	60.4	73.6
>12 years	74.1	52.8	47,0	57.7	55.3	65.2	45.5	45.5	74.8
*Paternal educational level*									
<10 years	44.5	39.3	45.6	48.2	41.1	51.6	42.4	58.5	47.2
10–12 years	58.2	46.5	45.8	52.4	59.6	54.1	51.9	60.8	71.6
>12 years	73.1	53.2	50.9	57.5	59.3	40.5	53.6	67.5	78.9
*Parental attachment to labour market*									
Mother in work	63.2	43.3	43.0	49.9	52.4	48.2	44.9	55.0	74.2
Mother out of work	51.5	41.8	47.0	50.6	49.8	51.0	45.5	58.6	58.0
Father in work	63.0	44.0	45.1	49.5	49.2	49.0	44.7	62.2	73.1
Father not in work	49.0	40.3	41.1	54.0	53.0	51.1	48.1	54.8	55.6
*Household income percentile*									
<6 %	50.6	37.1	55.0	41.7	51.9	47.4	36.7	55.2	54.6
6–24 %	47.2	41.7	45.5	44.3	50.9	49.5	50.6	56.8	64.4
25–49 %	54.6	44.5	44.1	53.0	41.4	48.7	50.0	79.0	69.0
50–74 %	64.9	51.0	40.6	54.7	72.7	50.0	0.0	57.1	68.5
75–100 %	74.0	68.0	45.5	63.6	66.7	60.0	50.0	50.0	81.8
*Maternal acculturation indicators*									
Migrated to Denmark		46.0	48.1	49.5	50.0	50.0	45.4	57.8	73.4
Descendent of migrants		31.6	39.7	52.1	50.0	46.3	36.4	n.e.	60.0
*Number of years lived in Denmark before delivery*									
<10 years		47.7	51.9	49.5	53.1	58.1	41.5	60.5	71.0
10+ years		44.9	42.3	49.5	38.7	44.4	46.7	50.0	77.3
*Age at migration to Denmark*									
<10 years		43.4	39.5	45.7	14.3	46.7	39.3	55.6	70.6
10–18 years		44.7	55.3	48.5	48.2	48.0	54.4	45.5	64.7
>18 years		49.9	48.7	51.5	53.5	52.8	38.5	65.0	74.1

The risk of suboptimal breastfeeding according to maternal country of origin and parental socio-demographic characteristics can be found in Table 3. Except for the Afghan minority, all non-Nordic minorities had statistically significant elevated risks compared to the Danish majority. The pattern of descendants having higher risk than migrants was found in the Turkish, Pakistani, Lebanese and Moroccan minority, but the difference did only reach statistically significance for the Turkish minority. Infants of mothers < 25 years had essentially the same significantly elevated risk of suboptimal breastfeeding, irrespective of country of origin. While the risk estimates for all strata of maternal age differed significantly from the reference group in all minority groups, the confidence intervals for the age-related risk estimates were overlapping within the minority groups. The same phenomenon was seen for maternal parity and all of the socio-economic variables.

**Table 3:** Crude odds ratios (95 % CI) for suboptimal breastfeeding^a^ according to socio-demographic characteristics and maternal country of origin

	Maternal country of origin									
	Denmark	Turkey	Pakistan	Former Yugoslavia		Iraq	Morocco	Lebanon/Palestine	Afghanistan	Other Nordic
*Migrant status*										
Overall	1 (ref)	2.17 (1.95–2.42)	1.93 (1.63–2.29)	1.61 (1.36–1.92)		1.62 (1.27–2.05)	1.67 (1.26–2.22)	1.99 (1.48–2.69)	1.18 (0.86–1.62)	0.64 (0.50–0.83)
Migrant		1.9 (1.68–2.14)	1.75 (1.43–2.13)	1.65 (1.36–2.01)		1.62 (1.27–2.06)	1.62 (1.18–2.23)	1.95 (1.43–2.65)	1.18 (0.86–1.62)	0.59 (0.44–0.78)
Descendants of migrants		3.5 (2.77–4.42)	2.46 (1.81–3.35)	1.49 (1.03–2.14)		1.62 (0.10–25.9)	1.87 (1.01–3.46)	2.83 (0.89–9.67)	n.e.	1.08 (0.57–2.03)
*Maternal age at birth*										
< 25 years	3.51 (3.20–3.85)	3.97 (3.20–4.92)	2.49 (1.69–3.67)	2.28 (1.58–3.29)		2.27 (1.41–3.67)	2.17 (1.02–4.63)	3.25 (1.99–5.32)	1.85 (1.04–3.30)	2.02 (0.93–4.36)
25–29 years	1.53 (1.43–1.63)	2.61 (2.20–3.10)	2.39 (1.87–3.06)	2.12 (1.62–2.75)		1.81 (1.20–2.73)	2.55 (1.58–4.13)	2.37 (1.36–4.14)	1.61 (0.94–2.77)	0.91 (0.52–1.59)
30–34 years	1 (ref)	2 (1.60–2.52)	2.49 (1.79–3.47)	1.52 (1.08–2.14)		2.21 (1.36–3.61)	1.68 (0.99–2.86)	1.7 (0.87–3.31)	1.21 (0.64–2.30)	0.75 (0.51–1.12)
35 + years	0.87 (0.82–0.94)	2.67 (1.65–3.12)	2.18 (1.26–3.76)	2.57 (1.45–4.49)		1.85 (1.04–3.30)	1.93 (1.06–3.51)	2.22 (0.94–5.23)	1.01 (0.41–2.50)	0.56 (0.33–0.94)
*Maternal parity*										
1	1 (ref)	1.97 (1.62–2.39)	1.56 (1.41–2.12)	1.6 (1.21–2.19)		1.41 (0.92–2.14)	1.86 (1.06–3.26)	2.69 (1.57–4.60)	1.39 (0.83–2.32)	0.73 (0.50–1.07)
2	0.73 (0.69–0.77)	1.78 (1.46–2.71)	1.66 (1.20–2.29	1.3 (0.96–1.75)		1.56 (0.96–2.53)	1.62 (0.94–2.79)	2.02 (0.97–4.18)	0.9 (0.48–1.69)	0.44 (0.27–0.71)
3+	0.65 (0.60–0.70)	1.63 (1.33–1.99)	1.42 (1.06–1.90)	0.9 (0.60–1.36)		1.1 (0.71–1.71)	1.14 (0.71–1.81)	0.91 (0.54–1.55)	0.67 (0.35–1.28)	0.57 (0.31–1.07)
*Maternal educational level*										
<10 years	4.86 (4.49–5.27)	4.1 (5.53–4.76)	4.43 (3.28–5.98)	3.6 (2.64–4.91)		3.24 (2.23–4.69)	3.63 (2.36–5.60)	4.36 (2.89–6.58)	1.98 (1.21–3.24)	3.16 (1.28–7.79)
10–12 years	2.23 (2.10–2.36)	3.55 (2.91–4.34)	3.35 (2.55–4.39)	2.86 (2.20–3.71)		2.78 (1.77–4.04)	2.86 (1.75–4.67)	2.19 (1.27–3.78)	1.87 (1.05–3.34)	1.03 (0.64–1.66)
>12 years	1 (ref)	2.56 (1.80–3.63)	3.22 (2.09–4.97)	2.1 (1.34–3.29)		2.31 (1.22–4.39)	1.52 (0.65–3.60)	3.43 (1.05–11.24)	3.43 (1.48–7.94)	0.96 (0.65–1.41)
*Paternal educational level*										
<10 years	3.4 (3.14–3.68)	4.21 (3.60–4.94)	3.26 (2.44–4.34)	2.93 (2.09–4.12)		3.9 (2.56–5.95)	2.56 (1.56–4.19)	3.7 (2.21–6.22)	1.93 (1.04–3.60)	3.04 (1.58–5.86)
10–12 years	1.96 (1.85–2.08)	3.14 (2.56–3.84)	3.22 (2.42–4.30)	2.47 (1.92–3.19)		1.85 (1.22–2.80)	2.31 (1.39–3.83)	2.53 (1.48–4.32)	1.76 (1.00–3.09)	1.08 (0.71–1.65)
>12 years	1 (ref)	2.39 (1.71–3.35)	2.62 (1.79–3.85)	2.01 (1.26–3.20)		1.87 (1.09–3.23)	3.99 (2.07–7.71)	2.36 (1.12–4.97)	1.31 (0.68–2.55)	0.73 (0.48–1.12)
*Parental attachment to labour market*										
Mother in work	1 (ref)	2.25 (1.95–2.60)	2.27 (1.75–2.96)	1.73 (1.39–2.14)		1.56 (0.85–2.86)	1.84 (1.20–2.82)	2.1 (1.20–3.70)	1.32 (0.71–2.47)	0.6 (0.45–0.80)
Mother out of work	1.61 (1.15–1.74)	2.39 (2.03–2.81)	1.93 (1.55–2.41)	1.68 (1.24–2.26)		1.73 (1.33–2.25)	1.65 (1.18–2.43)	2.06 (1.44–2.95)	1.14 (0.79–1.65)	1.24 (0.71–2.18)
Father in work	1 (ref)	2.17 (1.91–2.45)	2.07 (1.72–2.50)	1.73 (1.43–2.10)		1.75 (1.24–2.49)	1.77 (1.28–2.46)	2.1 (1.37–3.23)	1.03 (0.67–1.58)	0.62 (0.48–0.82)
Father out of work	1.79 (1.61–1.99)	2.52 (2.01–3.15)	1.63 (1.07–2.46)	1.45 (0.92–2.28)		1.51 (1.08–2.12)	1.63 (0.91–2.92)	1.84 (1.17–2.87)	1.4 (0.85–2.31)	1.36 (0.54–3.45)
*Household income percentile*										
<6 %	2.78 (2.38–3.25)	4.82 (3.48–6.68)	2.33 (1.49–3.63)	3.98 (2.05–7.74)		2.63 (1.52–4.55)	3.16 (1.28–7.79)	4.91 (2.33–10.34)	2.31 (1.11–4.81)	2.37 (0.72–7.78)
6–24 %	3.18 (2.92–3.46)	3.98 (3.40–4.66)	3.4 (2.58–4.78)	3.58 (2.64–4.85)		2.74 (2.01–3.74)	2.9 (1.97–4.26)	2.78 (1.83–4.23)	2.16 (1.44–3.25)	1.57 (0.85–2.90)
25–49 %	2.37 (2.20–2.55)	3.54 (2.86–4.39)	3.6 (2.61–4.97)	2.52 (1.88–3.39)		4.03 (1.92–8.45)	2.99 (1.59–5.62)	2.84 (1.48–5.48)	0.76 (0.25–2.29)	1.28 (0.73–2.24)
50–74 %	1.54 (1.43–1.65)	2.73 (1.85–4.05)	4.16 (2.52–6.86)	2.36 (1.49–3.73)		1.07 (0.28–4.02)	2.84 (1.23–6.57)	N.e.	2.13 (0.48–9.54)	1.31 (0.80–2.15)
75–100 %	1 (ref)	1.34 (0.58–3.11)	3.41 (1.47–7.91)	1.63 (0.80–3.31)		1.42 (0.26–7.77)	1.9 (0.32–11.35)	2.84 (0.18–45.48)	2.84 (0.18–45.43)	0.63 (0.38–1.06)

^a^Suboptimal breastfeeding: non-adherence to the Danish Health and Medicines Authority recommendation of exclusively breastfeeding at least to the age of 4 months

The risk of suboptimal breastfeeding was estimated on the subsample with full information on all co-variables. The estimated crude risk can be seen in Table 4 together with the estimated odds ratios adjusted for maternal age and parity, maternal and paternal education, maternal and paternal attachment to labour market and household income. The crude estimates were comparable to those estimated in the full sample (Table 3), but adjustment for the co-variables attenuated the estimated risks considerably, leaving all estimates, except the estimate for the Pakistani minority, close to unity. Infants with Pakistani origin had an adjusted odds ratio of 1.31 for being suboptimally breastfed and infants of Afghan origin has a decreased risk [OR 0.56 (95 % CI 0.36–0.87)] as compared to infant of Danish mothers.

**Table 4:** Odds Ratios for suboptimal breastfeeding^a^ according to maternal country of origin adjusted for socio-demographic characteristics^b^

	Maternal country of origin								
	Denmark	Turkey	Pakistan	Former Yugoslavia	Iraq	Morocco	Lebanon/Palestine	Afghanistan	Other Nordic
N = 23,596	21,103	1,028	369	375	183	129	112	95	202
*Full information on covariats*									
Crude OR	1 (ref)	2.09 (1.84–2.37)	1.95 (1.58–2.39)	1.52 (1.24–1.86)	1.38 (1.03–1.85)	1.76 (1.24–2.49)	1.75 (1.21–2.53)	0.95 (0.63–1.44)	0.7 (0.52–0.95)
Adjusted OR	1 (ref)	1.06 (0.92–1.22)	1.31 (1.06–1.63)	0.98 (0.79–1.21)	0.79 (0.58–1.08)	1.1 (0.77–1.57)	0.88 (0.60–1.30)	0.56 (0.36–0.87)	0.81 (0.59–1.12)

^a^Suboptimal breastfeeding: non-adherence to the Danish Health and Medicines Authority recommendation of exclusively breastfeeding at least to the age of 4 months

^b^Adjusted for maternal age, parity, maternal and paternal education, maternal and paternal attachment to labour market, and household income

**Fig. 1:**
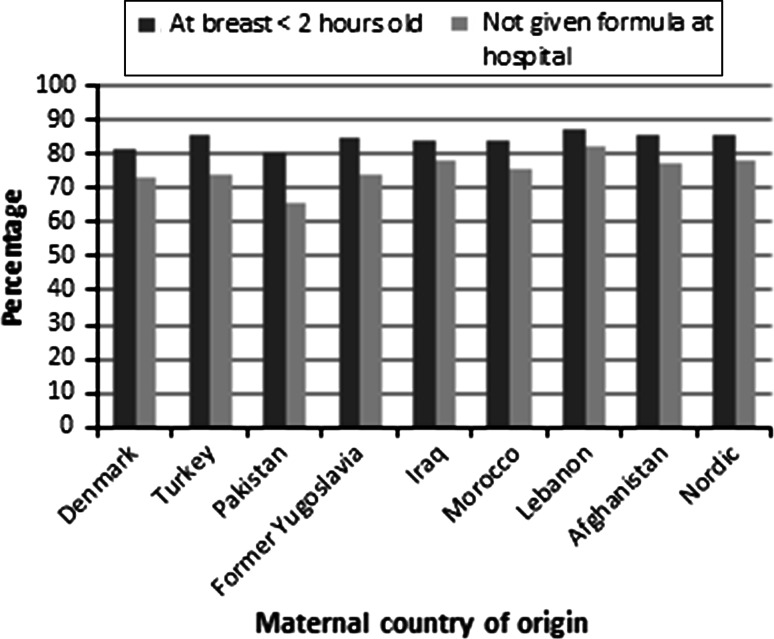
Distribution of indicators of breastfeeding support according to maternal country of orgin

The proportion of infants, who were exposed to the two indicators of breastfeeding support, can be seen in Fig. 1. From the figure follows that the proportions of infants that were put to the breast within 2 h of birth, and not being artificially fed (formula fed) at the hospital did, not differ according to maternal country of origin.

## Discussion

Suboptimal breastfeeding was more frequent among women of non-Nordic origin than among women with Danish origin. Among descendants of Turkish and Pakistani immigrants, the breastfeeding frequency further decreased, thus the acculturation process did not favor breastfeeding. For all minority groups, but the Pakistani, adjustment for socio-demographic variables abolished the increased risk of suboptimal breastfeeding. There were no differences between migrants and non-migrants in the breastfeeding support provided at hospital level.

Previous international studies are in general concluding that breastfeeding rates are higher among the migrant than the native populations [11–14]. It seems that this does not apply to the Danish context. This could be explained with the very high level of breastfeeding initiation in general in Denmark (95 %) relative to US (57 %) [11], UK (70 %) [12], and the Netherlands (90 %) [14]. Comparison of breastfeeding prevalence across countries is difficult because of differences in measures of duration and exclusivity of breastfeeding. However, in Denmark, around 46 % of women with Pakistani origin were fully breastfeeding at 4 months, while in a British context 36 % of women with Pakistani origin reported to be breastfeeding without introduction of any solid foods at 4 months [12]. Thus, the levels of breastfeeding in migrants in Denmark might not be different from the levels in other settings, and the gap to the women with Danish origin could be due to the high level in women of Danish origin.

Studies from the US have shown that the longer the migrant women have been in the country prior to birth, the lower breastfeeding prevalence, and further that descendants were breastfeeding less than immigrants [11]. The same pattern was seen for second generation Mediterranean migrants in the Netherlands, when compared to the first generation [14]. However, in Sweden, partial breastfeeding among migrants was the same among women, who had stayed less or more than 5 years [9], thus no negative effect of acculturation was indicated. In Denmark the negative effect of acculturation was seen in the crude analysis, however not in direction towards the native population, but towards increased disparity.

An anthropological study of perceptions of breastfeeding among women of Turkish origin in Denmark have found that the women had intentions to breastfeed, had initiated breastfeeding and had high knowledge about breastfeeding, but experienced early cessation [21]. The study elucidated that these women, in their process of acculturation were struggling to feel respected as modern women. To these women a modern life included to take part in the public spaces, but traditional norms made them consider breastfeeding as a practice only acceptable in domestic spaces. As a consequence, some women shortened breastfeeding duration. We speculate that this example of everyday life experiences of being between two cultures could be one example of many acculturation processes, and that these might explain the lower frequencies of optimal breastfeeding in women of non-Western origin in Denmark.

For the Danish-born women the breastfeeding frequency was higher among women in work than among women out of work, which we see as a result of the supportive Danish legislation for maternity leave. Among the migrants this tendency was less clear, except for the other Nordic immigrants, and this finding might reflect the contemporary weaker attachment to the Danish labour market among the migrant women.

Adjustment for maternal age, parity, and paternal education, employment status, and income abolished the increased risk of suboptimal breastfeeding in all groups, except the Pakistani. In a strict sense, these factors do not act as confounders, but may be mediators of the association demonstrated. In other words, the crude odds ratios do represent the true, not confounded, increased risk of suboptimal breastfeeding that offspring of non-Nordic mothers face. The results of the adjusted analyses indicate, however, that this increased risk is a result of the socio-economic circumstances women of non-Nordic origin in Denmark currently face. In the Nordic countries, suboptimal health care for migrants has been shown [22, 23] and migrants have reported discrimination in the Danish health care system [24]. However, in this study we found no indication of discrimination as there were no migrant specific differences in the breastfeeding support provided at hospital level.

### Study Limitations

The data available for this study was a unique combination of health visitor data and population covering registries. The health visitor data included information from more than 90 % of children born in the included 18 municipalities in the study period and thus most likely less subject to selection bias than other (often survey) studies of breastfeeding.

There was high occurrence of missing data on full breastfeeding, but it was reassuring regarding risk of biased estimates that no particular skewness was found the distribution of the co-variables between women with and without information on breastfeeding.

## Conclusions and Perspectives

Suboptimal breastfeeding was more frequent among women of non-Nordic origin than among women with Danish origin. Women, who were descendants of Turkish and Pakistani immigrants, had an even lower breastfeeding frequency than first generation migrants from these countries, indicating that the acculturation process did not favor breastfeeding. This finding is of public health relevance to the health professionals, who needs to be aware of vulnerability of these mothers. The negative effect of acculturation makes it relevant to further study the reasons for breastfeeding cessation among the Turkish and Pakistani women and a qualitative approached combined with richer and more detailed information on breastfeeding practices from survey data seems appropriate. Based on such studies strategies for how best to support these women, and the health of their infants/children, could be developed.
